# Managing soft tissue sarcomas in a developing country: are prognostic factors similar to those of developed world?

**DOI:** 10.1186/1477-7819-10-188

**Published:** 2012-09-13

**Authors:** Irfan Qadir, Masood Umer, Hafiz Muhammad Umer, Nasir Uddin, Farrok Karsan, Muhammad Sharoz Rabbani

**Affiliations:** 1 Orthopaedic Surgery Department, Aga Khan University Hospital, Faculty Offices opposite Community Health Centre, Aga Khan University Hospital, Karachi 74800, Pakistan; 2 Department of Pathology and Microbiology, Faculty Offices opposite Community Health Centre, Aga Khan University Hospital, Karachi 74800, Pakistan; 3 Department of Radiation Oncology, Faculty Offices opposite Community Health Centre, Aga Khan University Hospital, Karachi 74800, Pakistan; 4 Department of Surgery, Aga Khan University Hospital, Room 106, Male hostel, Karachi 74800, Pakistan

**Keywords:** Soft tissue sarcoma, Developing country, Pakistan, Local recurrence, Prognostic factors, Survival

## Abstract

**Background:**

Managing soft tissue sarcomas (STS) in a developing country with limited financial resources and a poor health referral system is a challenge. Presenting late, these extremity STS are prone to recurrence despite apparently complete resection. This study aimed to explore and compare the impact of clinico-pathological factors on recurrence and survival in Pakistan with the corresponding figures quoted from the developed world.

**Methods:**

An institutional review was performed on all patients with primary STS of the extremities operated on between 1994 and 2008. The prognostic influence of clinical, pathologic, and treatment variables on local recurrence free survival (LRFS), metastasis free survival (MFS) and overall survival (OS) were analyzed by univariate and multivariate Cox regression analysis and Kaplan Meier survival curves.

**Results:**

A total of 84 patients with a mean age of 41.8 ± 21.9 years were included in the study. The local recurrence rate was 14.3% after a median of 6 (mean 7.4) months. Metastases occurred in 7 patients (8.3%) and 65 patients were alive without evidence of disease after a mean follow-up of 52.6 ± 39.8 months. Tumor size > 5 cm, grade 3 tumors and margin < 10 mm significantly increased local recurrence rates. A margin ≥ 10 mm and age < 45 years significantly enhanced cumulative survival. Significant multivariate risk factors for metastases were margin < 10 mm and tumor grade G3.

**Conclusions:**

Despite a poor health referral system in our country, our results are no different from those reported from the developed world. Surgical margins and tumor grade prognostically influenced LRFS, MFS and OS.

## Background

Soft-tissue sarcomas (STS) form a large and heterogeneous group of mesenchymal extraskeletal malignancies that account for < 1% of all malignant tumors in the general population [1]. ST can develop virtually anywhere in the body, however, most tumors originate in an extremity (59%), the trunk (19%), the retroperitoneum (15%), or the head and neck (9%) [2]. Despite the variety of histologic subtypes, soft tissue sarcomas are grouped together at the clinical level because of parameters such as location, growth pattern and likelihood of recurrence, patient age, metastases, therapy, and prognosis [1].

Treatment of extremity STS has seen an evolution from radical surgery with liberal use of amputation to a limb-sparing approach [3]. The major therapeutic goals are long-term survival, avoidance of local recurrence, maximizing function and minimizing morbidity. Landmark trials conducted in the 1970s and 1980s at the National Cancer Institute showed equivalent survival outcomes between limb amputation and limb sparing surgery combined with radiotherapy (RT) [4,5]. However, despite apparently complete resections, one-third of patients with extremity STS suffer recurrence, typically within two years [6,7]. Several risk factors associated with recurrence have repeatedly been reported in the literature, including histologic subtype; tumor location, size, depth, and grade; and surgical margin [8-10].

Managing STS in a developing country is a challenge. Limited financial resources do not allow patients to come early to tertiary care centers. Presenting late, these extremity STS are prone to recurrence despite apparently complete resection. Against this background we hypothesized that patients from this part of the world would have a poorer prognosis with higher recurrence rates and lower overall survival. Given the reported geographic variance between prognostic factors for other cancers, we also hypothesized that similar variance would exist in STS as well and a different set of prognostic factors would influence recurrence rates and survival in our population. The goal of this investigation was to explore the impact of clinic-pathological factors on local recurrence-free survival (LRFS), distant metastasis-free survival (DMFS), and overall survival (OS) in patients with primary localized extremity STS undergoing surgical resection and compare it with figures quoted from the developed world since no similar studies have been presented from our country.

## Methods

This study was a retrospective cohort analysis of consecutive patients with STS of an extremity, who did not have synchronous metastasis or local recurrence (LR) on presentation, and who received primary surgery at the Aga Khan University Hospital from January 1994 through December 2008.

The demographic data and clinical characteristics of the study population were acquired from clinical chart review, tumor registry information, physicians’ records, patients’ correspondence, and telephone interviews.

All tumors were reviewed by experienced pathologists at our institution. Tumors were diagnosed and graded according to the FNCLCC (French Federation Nationale des Centres de Lutte Contre le Cancer) system [11]. For analyses, FNCLCC grade two and three tumors were defined as high grade tumors, and grade one as low grade [12]. Tumor size was classified as < 5 cm or ≥ 5 cm. Tumors were characterized as superficial or deep according to the involvement of the investing fascia [13].

Margins were evaluated intra-operatively by a dedicated pathologist. Margins were inked and separately sampled. The closest margin was microscopically categorized as positive (tumor within 1 mm of the inked surface) or negative (absence of tumor within 1 mm of the inked surface) and was further classified into the following categories: < 4 mm, 5 to 9 mm, 10 to 19 mm and ≥ 20 mm. Radiotherapy was recommended for patients with tumors exhibiting high risk factors for recurrence: anyone or combination of factors including size > 5 cm, high grade, deep tumors and inadequate surgical margins.

Approval for this study was sought from our hospital’s Ethical review Committee.

### Statistical analysis

Patients were evaluated from the time of their histological diagnosis up to their latest uneventful follow-up visit or disease progression, recurrence, metastasis or death. Patients who did not experience the event of interest or death over the course of the study were censored at their last follow-up. LRFS rates were calculated from diagnosis to local progression or relapse at the same or adjacent tumor site. All other tumor recurrences were classified as distant metastases and metastases-free survival (MFS) rates were calculated from diagnosis to the onset of distant metastases.

To explore the prognostic factors for the survival of patients, a univariate analysis with the Kaplan–Meier method was estimated for survival curves, and the log-rank statistical test with two-sided test was applied to test the significance between survival curves and every potential risk factor. Variables were included on the basis of previous reports from the literature. A stepwise multivariate Cox regression survival model was applied to evaluate the potential prognostic factors identified in the univariate analysis. The limits for the selection were *P* = 0.05 for entry and *P* = 0.10 for removal by the likelihood ratio test. Data were analyzed using SPSS software (version 19, SPSS, Chicago, IL, USA).

## Results

### Clinico-pathological characteristics of the patients

During the study period, a total of 84 consecutive adult STS patients received treatment at our institute. The main features of the study population and patient management are summarized in Table 1. The mean age was 41.8 ± 21.9 years. There were 46 men (54.8%) and 38 women (45.2%). Most patients presented with an asymptomatic mass (n = 60). Median duration of symptoms was 12 months (range: 1 to 174 months). The histological diagnosis of the tumor was established by biopsy. The samples were obtained in the clinic by making a stab incision and using a small curette to take the tissue for biopsy as recommended by National Comprehensive Cancer Network guidelines. The majority of tumors were located in the lower limb (70.2%). Pleomorphic liposarcoma, synovial cell sarcoma, spindle cell sarcoma and malignant fibrous histiocytoma (MFH) were the most common tumor types, accounting for 65.5% of all tumors. All tumors were STS and were classified according to FNCLCC (tumor differentiation, mitotic activity, and extent of necrosis). Most of the tumors were high grade (84.5%), ≥ 5 cm in size (59.5%) and deep-seated (44%). Eighteen (21.4%), 28 (33.3%), 34 (40.5%) and 4 (4.8%) were categorized as having margin widths of 1 to 4 mm, 5 to 9 mm, 10 to 19 mm and ≥ 20 mm respectively. Postoperative radiation was administered to 60 patients. Patients with prognostic factors indicating high risk for recurrence were recommended for radiation therapy. Radiation therapy was administered for G3 tumors in 27 patients, G2 tumors in 23 patients and G1 tumors in 10 patients. Radiation therapy was given to the entire surgical bed with 3 to 5 cm margin beyond the surgical scar and/or beyond post-operative seroma or areas of ecchymoses. The dose delivered was 50 Gy in 25 fractions using computed tomography (CT) based three dimensional conformal treatment planning. The scar was bolused to ensure full dose to the surface. The field size was then reduced to the primary surgical bed plus a 2 cm margin for a further dose of 10 Gy in 5 fractions. If the margin was microscopically positive a further 6 Gy in 3 fractions was delivered to that area for a total of 66 Gy in 33 fractions.

**Table 1:** Patient characteristics

**Characteristics**	**Total**	**Margin <10 mm**	**Margin >10 mm**	**P value**
	**Number = 84, No. (%)**	**Number = 46, No. (%)**	**Number = 38, No. (%)**	
Age				0.162
< 45	47	23 (50%)	24 (63.2%)	
≥ 45	37	23 (50%)	14 (76.8%)	
Gender				0.282
Male	46 (54.8%)	27 (58.7%)	19 (50%)	
Female	38 (45.2%)	19 (41.3%)	19 (50%)	
Tumor site				0.350
Upper	25 (29.8)	15 (32.6%)	10 (26.3%)	
Lower	59 (70.2%)	31 (67.4%)	28 (77.7%)	
Grade				0.041
1	13 (15.5%)	03 (6.5%)	10 (26.3%)	
2	31 (36.9%)	18 (39.1%)	13 (34.2%)	
3	40 (47.6%)	25 (54.3%)	15 (39.4%)	
Size				0.308
<5 cm	34 (40.5%)	17 (37%)	17 (44.7%)	
>5 cm	50 (59.5%)	29 (43%)	31 (55.3%)	
Radio				0.567
Yes	60 (71.4%)	27 (58.7%)	33 (86.8%)	
No	24 (28.6%)	19 (41.3%)	11 (13.2%)	
Margin				
1-4 mm	18 (21.4%)	-	-	-
5-9 mm	28 (33.3%)	-	-	-
10-19 mm	34 (40.5%)	-	-	-
>20	04 (4.8%)	-	-	-
Depth				0.111
Superficial	47 (56%)	29 (63%)	18 (47.4%)	
Deep	37 (44%)	17 (37%)	20 (52.6%)	
Recurrence				0.112
Yes	12 (14.3%)	09 (19.6%)	03 (7.9%)	
No	72 (85.7%)	37 (80.4%)	35 (92.1%)	
Metastasis				0.303
Yes	07 (8.3%)	05 (10.9%)	02 (5.3%)	
No	77 (91.7%)	41 (89.1%)	36 (94.7%)	
Status				0.006
Dead	08 (9.5%)	08 (17.4%)	00 (0%)	
Alive	76 (90.5%)	38 (82.6%)	38 (100%)	

### Patients’ outcome after primary surgery

The mean follow-up period (starting from the date of surgery) was 52.6 ± 39.8 months (median 36 months). Twelve patients (14.3%) developed LR at a mean of 7.4 months (median 6 months). Distant metastases occurred in seven patients (8.3%). At the end of the study, 65 patients were alive and without evidence of disease while eight patients died during this period.

Actuarial one year and three year LRFS rates were 87.9% and 85.4%, respectively. There were no local recurrences after three years. The Kaplan–Meier freedom from distant metastases for all patients was estimated to be 95.2% at one year, 94% at two years and 92.8% at three years. There were no distant metastases three years post operatively. Actuarial one year, two year and three year OS rates were 95.2%, 94% and 92.9% respectively.

### Prognostic factors influencing survival

Table 2 and Table 3 summarize the independent prognostic factors for survival by univariate and multivariate analyses respectively. As expected, higher grade tumor, large size tumor, and margins < 10 mm were identified as adverse factors associated with worse LRFS, MFS, and OS.

**Table 2:** Survival analysis according to univariate Cox proportional hazard models

**Characteristics**	**LRFS**			**MFS**			**OS**		
	**CHR**	**95% CI**	**P**	**CHR**	**95% CI**	**P**	**CHR**	**95% CI**	**P**
**Age**									
< 45 vs. ≥ 45	1.66	1 to 2.98	0.05	1.27	0.91 to 1.78	0.157	1.02	1.02 to 1.03	0.01
**Size**									
< 5 cm vs. ≥ 5 cm	2.70	1.97 to 3.81	<0.001	2.47	1.12 to 5.51	0.026	1.74	1.16 to 2.63	0.025
**Depth**									
Superficial vs. Deep	1.24	0.78 to 1.95	0.363	1.46	0.82 to 2.6	0.198	1.32	0.92 to 1.88	0.130
**Grade**									
Low vs. High	2.1	1.4 to 3.4	<0.001	3.45	1.63 to 7.373	<0.001	1.28	1.01 to 1.63	0.04
**Margin**									
< 10 mm vs. ≥ 10 mm	6.344	3.01 to 13.37	<0.001	3.98	2.2 to 7.11	<0.001	4.6	2.6 to 8.4	<0.001

*CHR*, Cox Hazard Ratio; *CI*, confidence interval; *LRFS*, local recurrence-free survival; *MFS*, metastasis-free survival; *OS*, overall survival.

**Table 3:** Survival analysis according to multivariable Cox proportional hazard models

**Characteristics and Survival endpoints**	**CHR**	**95% CI**	**P value**
**Factors associated with local recurrence-free survival**			
Size ≥ 5 cm	2.84	2.07 to 3.96	<0.001
Grade	2.2	1.1 to 4.5	0.024
Margin	3.88	1.52 to 9.90	<0.001
**Factors associated with metastasis-free survival**			
Grade	2.10	1.33 to 3.30	0.003
Margin	3.69	1.85 to 7.44	<0.001
**Factors associated with overall survival**			
Age	1.02	1.01 to 1.03	0.01
Margin	2.4	1.2 to 4.5	0.008

*CHR*, Cox Hazard Ratio; *CI*, confidence interval.

Among the 12 patients who had LR, 9 had grade 3 tumors and 10 of the primary tumors were larger than 5 cm. Kaplan Meier curves for patients with a tumor larger than or equal to 5 cm were found to be significantly different from curves for patients with a tumor smaller than 5 cm (*P* < 0.001). Estimated freedom from local recurrence rates for patients with a tumor larger or equal to 5 cm are 80% versus 94.1%.

Six out of seven patients with distant metastasis had G3 tumors and five had tumors larger than 5 cm. In addition, estimated freedom from distant metastases was significantly longer for patients having G1 and G2 tumors at initial diagnosis than for patients having G3 tumors (*P* < 0.001). The lungs (62.5%) were the most common site for initial presentation of distant metastasis.

**Figure 1:**
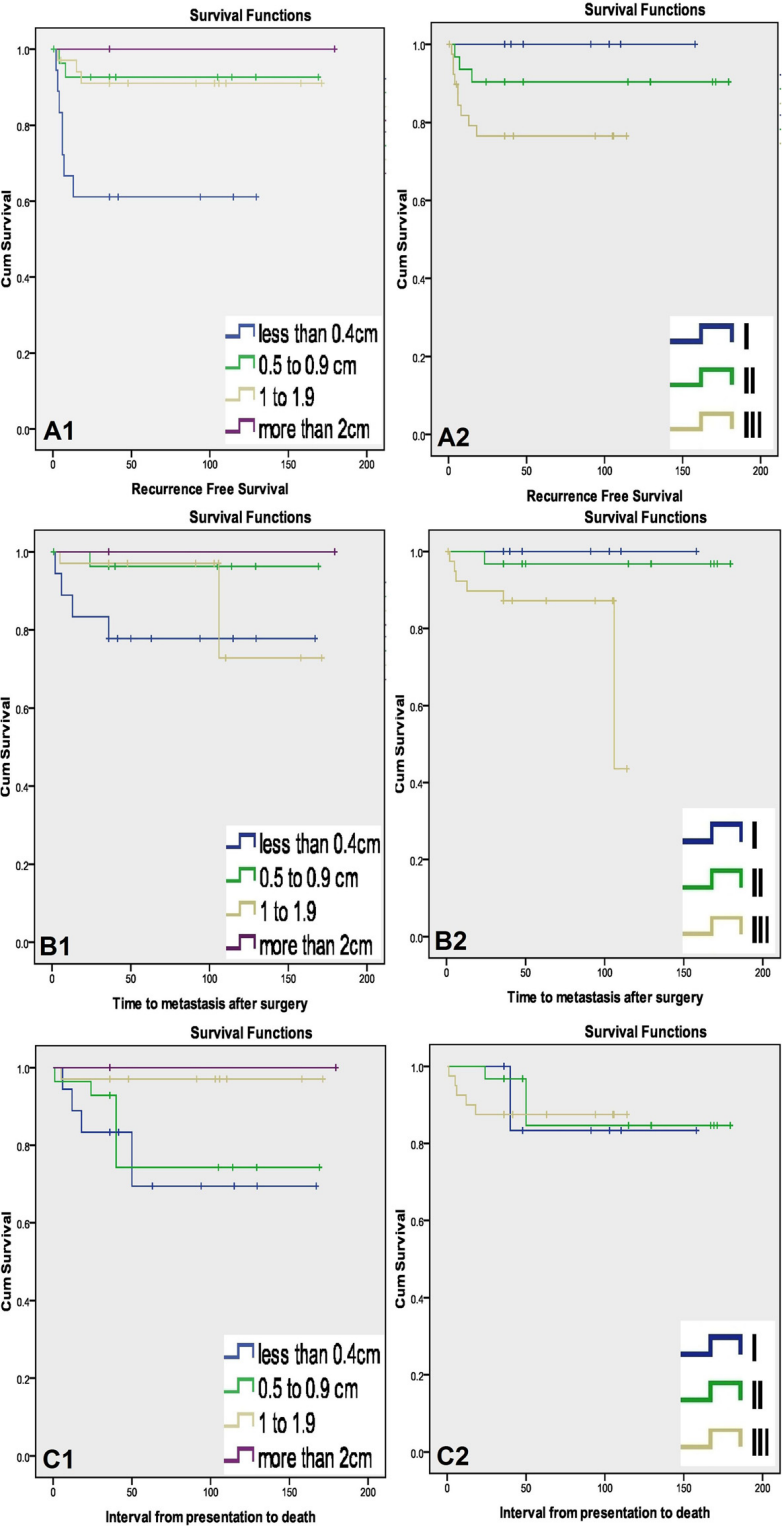
**(A) Local recurrence-free survival curves plotted according to 1: margin widths, 2: tumor grade.** (**B**) Distant metastasis-free survival curves plotted according to 1: margin widths, 2: tumor grade. (**C**) Overall survival curves plotted according to 1: margin widths, 2: tumor grade.

Since margin status was closely associated with local recurrence, the prognostic importance of margin width on LRFS, MFS and OS was estimated by the Kaplan–Meier method. As shown in Figure 1, a significant difference was found between groups. Therefore, an adequate negative margin for primary surgery was defined as a margin width ≥ 10 mm.

## Discussion

Soft tissue sarcomas are prone to recurrence. Literature has shown that despite apparently complete resections, one-third of patients with extremity STS suffer recurrence, typically within two years of the primary surgery [6,7]. Sarcomas tend to grow along muscle bundles and along fascial septa, sometimes well beyond the boundaries of the palpable tumor mass, and this characteristic at least partly explains the high frequency of local tumor recurrence after limited, or even wide, excision of soft-tissue sarcomas. Two of the most important clinico-pathological features that determine the likelihood of local control are anatomical extent of the sarcoma and whether the tumor was primary or recurrent [6]. It is important for clinicians to be aware that, in many of the more common types of STS (myxoid liposarcoma and myxofibrosarcoma), tumors may advance in grade in any recurrence, thereby increasing the risk of metastasis [6].

Since the development of limb salvage treatment, tumor size, depth, histologic grade, and anatomic site have been well-accepted prognostic factors affecting local recurrence and survival in patients with STS [8-10].

Pakistan is a third world country and its population is different from others in terms of access to health centers, awareness about the disease and socioeconomic status. There are only a few specialized cancer care centers in the country with a multidisciplinary approach towards these rare STSs. Patients, therefore, end up going to the wrong type of health care personnel and to getting the lesion excised without keeping in mind the suspicion of malignancy and safe margins. By the time they present to the specialized cancer care centers they have a poor prognosis. The presence of a multidisciplinary tumor board is an integral part of multimodal therapy for malignant diseases, particularly for sarcoma [3]. Ours is perhaps the only university hospital in the country which holds a monthly musculoskeletal tumor board meeting. A significant effort is made to evaluate all cases and come to a consensus level of management with regard to multi-modal therapy. Our hospital also maintains a musculoskeletal tumor registry. This registry not only enables our hospital to evaluate its cancer workload and the quality of medical care provided to patients but also serves as a resource for the continuing education of physicians and a stimulus for research by highlighting areas which require further study.

In this study, only those patients were included who came for primary surgery to our institute so that true prognostic factors could be identified. All cases presenting with a previous surgery outside our institute or a recurrence were excluded. We chose this population because no study had been done on them previously. Margin size, tumor grade, tumor-size, tumor depth and age of the patient were included in our study as prognostic factors.

The role of surgical margins has been extensively studied in recent years. Tumor resection for negative margins is the goal of surgical treatment for STSs. The literature has suggested that margin size has a strong correlation with the LRFS and disease specific survival (DSS) [8]. However, most of the studies only defined the margin as either positive (microscopic residual disease) or negative (no residual disease) [9,13-16]. Liu et al. [8] suggested 10 mm margin width as the critical threshold in identifying ‘adequate margin’. In contrast, the individual anatomy of the upper extremities, in particular of the hand, leads to an intentional reduction of resection margins in order to preserve the extremity and its function with the main intention of tumor-free resection margins [17]. On the other hand, authors such as Gronchi et al. [14] failed to confirm the prognostic influence of margin status on DSS in their study. This could be due to a higher number of liposarcomas in their study in which there are higher chances of achieving negative margins [14]. In this study, we found that margin size had a significant influence on LRFS, MFS and OS.

We also found that high grade tumors had higher risks of inadequate margins compared with low grade tumors [8]. This finding was consistent with a study carried out by Liu et al. Tumor depth has also been reported to be associated with local failure as well as poor over all prognosis. However, we failed to confirm the prognostic significance of tumor depth in our study, similar to results reported by Peiper et al. [18].

The significance of advanced age has been highlighted in several studies. Older age has been reported to be associated with lower survival rates in patients with STS [13,14,19]. Older patients tend to present with larger and higher grade tumor which possibly result in increased local recurrences [20]. Treatment variables can also be implicated as potential confounders for the effect of age, with a lower proportion of the elderly having definitive surgery or receiving chemotherapy [20] and radiotherapy [21]. Another study showed that the proportion of positive margins increased progressively with age [22]. All these factors can lead to a poor prognosis for patients more than 45 years old. In this study age had significant effect on overall survival; however we failed to confirm its prognostic influence on LRFS and MFS. Similar findings have been reported by Salo et al. [23] and Atalar et al. [24].

According to recommendations of the National Comprehensive Cancer Network guidelines, RT has been included in the paradigm to maintain excellent local control for high grade tumors although trials have failed to demonstrate a survival benefit. However, for low-grade tumors, observation is recommended unless there are close margins [25]. Schreiber *et al*. [26] tested the impact of postoperative radiation on OS for high-grade STS of the extremities on the Surveillance, Epidemiology and End Results (SEER) database and concluded that the use of postoperative radiation after radical limb sparing excision is associated with an improved OS and DSS only for patients with tumors >5 cm. In our study, radiotherapy was recommended for patients with tumors exhibiting high risk factors for recurrence. The retrospective nature of this study and the inherent selection bias limit conclusions with regard to the effectiveness of radiotherapy in maintaining local control.

Pre-operative radiotherapy, adjuvant and neo-adjuvant chemotherapy are not included in the standard treatment protocol for STS. These treatment modalities may be used when the tumor is of borderline operability and pre-operative radiotherapy/chemotherapy is judged to be capable of rendering the tumor operable. Adjuvant chemotherapy may be considered in situations where local relapse would be untreatable or where adequate radiotherapy could not be administered owing to the sensitivity of adjacent structures (class 2B evidence). In this study, chemotherapy was not used routinely but on a case by case basis if it was thought useful to spare the limb and often in combination with radiotherapy [27].

This study, however, is limited by the large time span and its retrospective design, which may result in temporal differences in factors such as patient profile, disease characteristics and treatment protocols and follow-up decisions. However in our data, year-wise split did not show such variance.

## Conclusions

We conclude that tumor size, tumor grade and margin status had a prognostic influence in our patients. Despite the lack of facilities and few multidisciplinary cancer care centers for these rare sarcomas, our results were no different from the data published from the developed world.

## Abbreviations

DMFS: Distant metastasis free survival; LRFS: Local recurrence free survival; MFS: Metastasis free survival; MFH: Malignant fibrous histiocytoma; NCCN: National Comprehensive Cancer Network; OS: Overall survival; RT: Radiotherapy; STS: Soft tissue sarcoma.

## Competing interests

The authors declare that they have no competing interests.

## Authors’ contributions

IQ and MU conceived the study and were involved in the literature review and manuscript preparation. HMU and SR were involved in data collection and performed statistical analysis. NU reviewed histology slides of all the patients for appropriate grading and margins. FK was involved in patient care and revised the manuscript. All authors read and approved the final manuscript.

## References

[B1] Lachenmayer A Yang Q Eisenberger CF Boelke E Poremba C Heinecke A Ohmann C Knoefel WT Peiper M Superficial soft tissue sarcomas of the extremities and trunk World J Surg 2009 33 1641 1649 10.1007/s00268-009-0051-1 19430830

[B2] DeVita VT Jr Hellman S Rosenberg SA Cancer: Principles and Practice of Oncology 2001 6 Lippincott Williams & Wilkins, Philadelphia 1841 1891

[B3] Wasif N Tamurian RM Christensen S Do L Martinez SR Chen SL Canter RJ Influence of specialty and clinical experience on treatment sequencing in the multimodal management of soft tissue extremity sarcoma Ann Surg Oncol 2012 19 504 510 10.1245/s10434-011-1923-9 21769468

[B4] Rosenberg SA Tepper J Glatstein E Costa J Baker A Brennan M DeMoss EV Seipp C Sindelar WF Sugarbaker P Wesley R The treatment of soft-tissue sarcomas of the extremities: prospective randomized evaluations of (1) limb-sparing surgery plus radiation therapy compared with amputation and (2) the role of adjuvant chemotherapy Ann Surg 1982 196 305 315 10.1097/00000658-198209000-00009 7114936 PMC1352604

[B5] Yang JC Chang AE Baker AR Sindelar WF Danforth DN Topalian SL DeLaney T Glatstein E Steinberg SM Merino MJ Rosenberg SA Randomized prospective study of the benefit of adjuvant radiation therapy in the treatment of soft tissue sarcomas of the extremity J Clin Oncol 1998 16 197 203 9440743 10.1200/JCO.1998.16.1.197

[B6] Singer S Demetri GD Baldini EH Fletcher CD Management of soft-tissue sarcomas: an overview and update Lancet Oncol 2000 1 75 85 10.1016/S1470-2045(00)00016-4 11905672

[B7] Alderman AK Kim HM Kotsis SV Chung KC Upper-extremity sarcomas in the United States: analysis of the surveillance, epidemiology, and end results database, 1973–1998 J Hand Surg Am 2003 28 511 518 10.1053/jhsu.2003.50076 12772113

[B8] Liu CY Yen CC Chen WM Chen TH Chen PC Wu HT Shiau CY Wu YC Liu CL Tzeng CH Soft tissue sarcoma of extremities: the prognostic significance of adequate surgical margins in primary operation and reoperation after recurrence Ann Surg Oncol 2010 17 2102 2111 10.1245/s10434-010-0997-0 20217247

[B9] Lahat G Tuvin D Wei C Anaya DA Bekele BN Lazar AJ Pisters PW Lev D Pollock RE New perspectives for staging and prognosis in soft tissue sarcoma Ann Surg Oncol 2008 15 2739 2748 10.1245/s10434-008-9970-6 18521685

[B10] Billingsley KG Lewis JJ Leung DH Casper ES Woodruff JM Brennan MF Multifactorial analysis of the survival of patients with distant metastasis arising from primary extremity sarcoma Cancer 1999 85 389 395 10.1002/(SICI)1097-0142(19990115)85:2<389::AID-CNCR17>3.0.CO;2-J 10023707

[B11] Trojani M Contesso G Coindre JM Rouesse J Bui NB de Mascarel A Goussot JF David M Bonichon F Lagarde C Soft-tissue sarcomas of adults; study of pathological prognostic variables and definition of a histopathological grading system Int J Cancer 1984 33 37 42 10.1002/ijc.2910330108 6693192

[B12] Mariani L Miceli R Kattan MW Brennan MF Colecchia M Fiore M Casali PG Gronchi A Validation and adaptation of a nomogram for predicting the survival of patients with extremity soft tissue sarcoma using a three-grade system Cancer 2005 103 402 408 10.1002/cncr.20778 15578681

[B13] Stojadinovic A Leung DH Hoos A Jaques DP Lewis JJ Brennan MF Analysis of the prognostic significance of microscopic margins in 2,084 localized primary adult soft tissue sarcomas Ann Surg 2002 235 424 434 10.1097/00000658-200203000-00015 11882765 PMC1422449

[B14] Gronchi A Casali PG Mariani L Miceli R Fiore M Lo Vullo S Bertulli R Collini P Lozza L Olmi P Rosai J Status of surgical margins and prognosis in adult soft tissue sarcomas of the extremities: a series of patients treated at a single institution J Clin Oncol 2005 23 96 104 15625364 10.1200/JCO.2005.04.160

[B15] Stojadinovic A Leung DH Allen P Lewis JJ Jaques DP Brennan MF Primary adult soft tissue sarcoma: time-dependent influence of prognostic variables J Clin Oncol 2002 20 4344 4352 10.1200/JCO.2002.07.154 12409334

[B16] Stoeckle E Gardet H Coindre JM Kantor G Bonichon F Milbeo Y Thomas L Avril A Bui BN Prospective evaluation of quality of surgery in soft tissue sarcoma Eur J Surg Oncol 2006 32 1242 1248 10.1016/j.ejso.2006.05.005 16793237

[B17] Lehnhardt M Hirche C Daigeler A Goertz O Ring A Hirsch T Drucke D Hauser J Steinau HU Soft tissue sarcoma of the upper extremities. Analysis of factors relevant for prognosis in 160 patients Chirurg 2012 83 143 152 10.1007/s00104-011-2124-6 21695557

[B18] Peiper M Zurakowski D Knoefel WT Izbicki JR Malignant fibrous histiocytoma of the extremities and trunk: an institutional review Surgery 2004 135 59 66 10.1016/S0039-6060(03)00325-8 14694301

[B19] Biau DJ Ferguson PC Turcotte RE Chung P Isler MH Riad S Griffin AM Catton CN O'Sullivan B Wunder JS Adverse effect of older age on the recurrence of soft tissue sarcoma of the extremities and trunk J Clin Oncol 2011 29 4029 4035 10.1200/JCO.2010.34.0711 21931025

[B20] Lahat G Dhuka AR Lahat S Lazar AJ Lewis VO Lin PP Feig B Cormier JN Hunt KK Pisters PW Pollock RE Lev D Complete soft tissue sarcoma resection is a viable treatment option for select elderly patients Ann Surg Oncol 2009 16 2579 2586 10.1245/s10434-009-0574-6 19557478

[B21] Al-Refaie WB Habermann EB Dudeja V Vickers SM Tuttle TM Jensen EH Virnig BA Extremity soft tissue sarcoma care in the elderly: insights into the generalizability of NCI Cancer Trials Ann Surg Oncol 2010 17 1732 1738 10.1245/s10434-010-1034-z 20354801

[B22] Trovik CS Bauer HC Alvegard TA Anderson H Blomqvist C Berlin O Gustafson P Saeter G Walloe A Surgical margins, local recurrence and metastasis in soft tissue sarcomas: 559 surgically-treated patients from the Scandinavian Sarcoma Group Register Eur J Cancer 2000 36 710 716 10.1016/S0959-8049(99)00287-7 10762742

[B23] Salo JC Lewis JJ Woodruff JM Leung DH Brennan MF Malignant fibrous histiocytoma of the extremity Cancer 1999 85 1765 1772 10.1002/(SICI)1097-0142(19990415)85:8<1765::AID-CNCR17>3.0.CO;2-K 10223571

[B24] Atalar H Basarir K Yildiz Y Saglik Y Prognostic factors in patients with malignant fibrous histiocytoma of the extremities Acta Orthop Traumatol Turc 2007 41 271 276 18180555

[B25] National Comprehensive Cancer Network guideline for soft tissue sarcoma V.1.2010 Available at: http://www.nccn.org/professionals/physician_gls/PDF/sarcoma.pdf 10.6004/jnccn.2010.0049 20581298

[B26] Schreiber D Rineer J Katsoulakis E Sroufe RL Lange CS Nwokedi E Schwartz D Choi K Rotman M Impact of postoperative radiation on survival for high-grade soft tissue sarcoma of the extremities after limb sparing radical resection Am J Clin Oncol 35 13 17 21278563 10.1097/COC.0b013e3181fe46d4

[B27] Grimer R Judson I Peake D Seddon B Guidelines for the Management of Soft Tissue Sarcomas Sarcoma 2010 506182 Epub 2010 May 31 10.1155/2010/506182 PMC2903951 20634933

